# Providing contextually apt feedback in clinical education

**DOI:** 10.5116/ijme.5ad7.b2b5

**Published:** 2018-05-04

**Authors:** Farshad Shaddel, Katy Newell-Jones, Denis O'Leary

**Affiliations:** 1 Department of Psychiatry, Medical Science Division, University of Oxford, UK; 2 School of General Practice (GP), Health Education England Thames Valley, UK; 3 Medical Sciences Division, University of Oxford, UK

**Keywords:** Contextually, apt feedback, clinical education

## Introduction

The importance of feedback in medical education is well established.^1^ There are several models of how to provide feedback effectively available to trainers. Each possesses innate strengths and weaknesses. For example, some are more trainer-centered and directive (e.g., the Sandwich model^2^) while others (e.g., the Pendleton^3^ and SET-GO^4^ models) promote learner-centeredness, reflection and explicit action planning respectively. Finkelstein and Fishbach,^5^ Rock,^6^ Shah & Higgins^7^ and Tuckman^8^ each suggest how trainers could adapt feedback conversations to specific trainer-trainee learning contexts. These papers can be separated into four categories based on the salient features of feedback conversations within, those contexts.

The first group of papers considers a ‘trainee’s level of competency and expertise’ and recommends how our feedback should be adapted as a result. For example, Finkelstein and Fishbach^5^ suggest that a more competent and expert trainee will respond better to negative feedback and greater challenge while a more novice trainee will respond to positive feedback and support.

The second group of papers considers the import of ‘trust and rapport between trainer and trainee’ on feedback effectiveness. David Rock discussed this in his SCARF model.^6^ When a trainee is relatively new and unknown to us, he recommends that trainers establish trust and rapport and explore the trainee’s interests before providing our feedback. In contrast, the model implies that trainers can potentially provide even quite challenging feedback promptly and frankly to a trainee with whom they have a good rapport and a trusting relationship.

The ‘purpose of feedback’ is the third contextual factor identifiable in the literature. If a learner does not adhere to required policies and professional regulations, Higgins & Higgins^7^ suggest that feedback emphasising the negative consequences of same to themselves and others could avoid repetition of the undesired behaviour. In contrast, feedback which emphasises the advantages and rewards of an action is more likely to inspire learners towards desirable performance.

Fourthly, the literature recommends trainers to be mindful of whether the feedback is provided to an individual alone or within a group. One-to-one settings can provide a safe space for any, including regulatory, feedback conversation. When giving feedback to a trainee in a group setting, trainers should consider the group’s stage of formation^8^ and norms, and the vulnerability to perceived criticism of that individual group member. Nevertheless, group members can provide both positive and challenging inspirational feedback to each other when group dynamics allow. In addition to being beneficial to each group member, it improves their skills in giving and receiving feedback.

These four contextual factors have been treated separately in the literature. In practice we have noted how the situational context is more complex; any combination of them may occur within a single trainer/trainee feedback conversation. For example, all four are present when an inexperienced trainee discusses their clinical cases during one-to-one supervision with their new clinical supervisor. We have noted how trainers faced with such situational complexity (and with limited time available to them) may come to rely on one or two feedback models with which they are more familiar. Not only does this reduce the effectiveness of our feedback, but it also lacks learner-centeredness as the model chosen is mainly based on what suits the trainer rather than the trainee.

This paper reports on how we developed some practical guidance for trainers on how to quickly formulate a feedback conversation that is responsive to the complex situational context in which that conversation occurs.

### Overview of the approach

Having identified these four “primary” (so referred to here for ease of communication) contextual factors, we have converted them into descriptive pairs that cover the breadth of each factor pragmatically - Novice (N) vs. Expert (E); Low (L) vs. High (H) rapport; Regulatory (R) vs. Inspirational (I) purpose; and One-to-one (O) vs. Group setting (G).  To describe a situational context that is inclusive of all four primary factors, we adopted the convention of listing the factors in the above order and selecting the first letter of each factor pair judged to be apt for that context. For example, we described as NLIO, the scenario mentioned earlier of an inexperienced trainee (N) discussing their cases at a one-to-one (O) supervision session with their new clinical supervisor (L) - the session being formative and inspirational (I) rather than regulatory. Using another example, we described as EHRO the context where a trainer has a one-to-one discussion (O) about adherence to professional norms (R) in a trusting relationship (H) with a senior trainee (E). Using this descriptive approach, there emerged 16 educational contexts derived from the original four primary contextual factors

In the next step, we constructed specific guidance tailored to each of the 16 educational contexts. The feedback content was constructed by combining the recommendations made in the literature for each of the four primary contextual factors and moulding it to their specific combination. For the two educational contexts already discussed (NLIO and EHRO), the guidance so derived was as follows:

#### NLIO

Open the conversation with exploratory questions to establish some rapport and get to know the learner, their competency level, insight, and inspiration. Then acknowledge the challenges they face when starting a new endeavour and provide positive and supportive feedback. In addition, it is important to emphasise the benefits of change to the learner and to check their understanding of any action plan and the supports available to them.

##### EHRO

Begin the conversation by sharing your observations on the learner’s performance and then ask for their thoughts. Explore with them the importance of following rules, guidelines, and policies, being clear about the “must do” elements and the consequences of deviating from them. Ensure that there is clarity about what is expected from the learner and the required action plan. And finally, mention the availability of an appeal process if the learner finds the feedback unfair.

These two examples demonstrate how tailoring feedback to the educational context can have an obvious impact on the content of that feedback conversation.  In a further refinement, we then sought to match an existing feedback model, based on its characteristics, with each of the 16 educational contexts. In this way, the Pendleton^3^ and SET-GO^4^ models respectively emerged as the most appropriate for the NLIO and EHRO contexts already cited. For completeness, we have created a matrix that summarises the guidance across the 16 educational contexts. This is available on request from the corresponding author.

## Conclusions

We have noted several challenges and considerations when using this guidance. Firstly, we should not assume that providing feedback is useful in every circumstance. Secondly, we adopted a narrower definition of feedback, considering it as a teaching tool while being mindful that recent papers^9^ tend to define feedback as a process; where the teacher should focus more effort on strengthening the learner’s skills of self-assessment and self-directed learning. Whilst being mindful of the importance of feedback as a process, in our experience clinical education has an additional element – the tension between self-directed learning on the one hand and minimum performance requirements in the interest of patient safety on the other.^10^ Consequently, there are many occasions in clinical education when we as trainers need to adopt an interventionist and directive approach to providing feedback. In other words, to use feedback as a tool, as adopted in this article. We have observed that utilising our guidance could facilitate a better outcome on such occasions.

In our approach, we have considered all four contextual factors as categorical while being mindful that degrees of expertise and relatedness are dimensional.  In this way, we aim to provide greater simplicity and clarity in the guidance.  In practice, we continue to judge position along both dimensions and use orientation towards either pole as an anchor to guide feedback delivery. Finally, we have considered only four primary contextual factors and three models of feedback delivery to produce the guidance. This reflects the limitations of a new point of departure and is not to be interpreted to the exclusion of other relevant factors and feedback models.

While acknowledging these challenges, to the best of our knowledge this is the first published guidance on facilitating contextually apt feedback within clinical education. Based on our experience using this guidance, the implications for other trainers include a more selective approach to feedback based on context with improved prospects of trainee receptiveness, development, and patient care. The immediate implication is that trainers familiarise themselves with and use the guidance. This will involve time input and practice initially. We anticipate that the more informal/conversational style and direct appeal to trainers within this article will facilitate this along with the additional information available on request. It signposts the trainer on how to respond to the contextual and dynamic complexity of the trainer-trainee relationship as well as our professional responsibility to protect patient safety. In this respect, it is unique within the medical education literature on feedback guidance. The more academic implications include further assessment of the reliability and validity of the guidance and its evaluation in the delivery of improved feedback, trainee performance, and patient care.

### Conflict of Interest

The authors declare that they have no conflict of interest.
